# THE EMED TOOLKIT: COLLABORATION TO IMPROVE ELDER MISTREATMENT SCREENING AND RESPONSE

**DOI:** 10.1093/geroni/igad104.0424

**Published:** 2023-12-21

**Authors:** Kristin Lees Haggerty, Rebecca Stoeckle, Kim Dash, Gary Epstein-Lubow, Randi Campetti, Ruthann Froberg, Ellen Tambor

**Affiliations:** Education Development Center, Inc., Waltham, Massachusetts, United States; Education Development Center, Inc., Waltham, Massachusetts, United States; Education Development Center, Waltham, Massachusetts, United States; Education Development Center, Waltham, Massachusetts, United States; Education Development Center, Waltham, Massachusetts, United States; Education Development Center, Inc., Waltham, Massachusetts, United States; Education Development Center, Waltham, Massachusetts, United States

## Abstract

The National Collaboratory to Address Elder Mistreatment (The Collaboratory) is a group of elder mistreatment (EM) researchers, clinicians, and advocates who came together with the goal of improving elder mistreatment identification and response in hospital emergency departments (ED). Since its inception in 2017, the Collaboratory has prioritized working in close collaboration with health care and aging services providers as well as older adult survivors to develop, test, and disseminate the Elder Mistreatment Emergency Department Toolkit (EMED Toolkit). The EMED Toolkit is a set of streamlined tools designed to improve EM screening and response in hospital EDs and foster improved collaboration between the hospital and community-based services. The EMED Toolkit is comprised of four core components 1) an online staff survey to assess EM practice and drive system change 2) the evidence-based EM Screening and Response Tool designed to efficiently screen all older adult patients for potential mistreatment, 3) online training modules to educate ED staff on elder mistreatment identification and response, including use of the screening tool, and 4) a Community Connections Roadmap designed to help EDs identify and establish connections with community-based services that can support safe discharge and provide ongoing support to older adults experiencing and at risk for mistreatment. In this presentation we will summarize results from a feasibility study testing EMED Toolkit implementation at five hospitals in the US, describe the Collaboratory’s approach to disseminating the EMED Toolkit more widely through local, regional, and national systems change initiatives, and share lessons learned from our efforts.

